# Neuropathologic and transcriptomic analysis reveals abnormal stress response in sporadic progressive supranuclear palsy autopsy brain tissue and human iPSC‐derived midbrain organoids

**DOI:** 10.1002/alz.093229

**Published:** 2025-01-03

**Authors:** Kristen R. Whitney, Margaret M. Krassner, Grace A. Selecky, Diana K. Dangoor, Claudia De Sanctis, Victoria Flores Almazan, Alessandra Cervera, Sean Thomas Delica, SoongHo Kim, Kurt W. Farrell, Abhijeet Sharma, Thomas D. Christie, Megan A Iida, Hadley A. Walsh, Lily Sarrafha, Gustavo Parfitt, Ruth H. Walker, Giulietta M. Riboldi, Steven J. Frucht, Tim Ahfeldt, Bin Zhang, Ana C. Pereira, Sally Temple, John F. Crary, Won‐min Song

**Affiliations:** ^1^ Ronald M. Loeb Center for Alzheimer’s Disease, New York, NY USA; ^2^ Icahn School of Medicine at Mount Sinai, New York, NY USA; ^3^ Friedman Brain Institute, New York, NY USA; ^4^ Icahn School of Medicine at Mount Sinai, New York, CA USA; ^5^ Department of Artificial Intelligence & Human Health, Nash Family Department of Neuroscience, Ronald M. Loeb Center for Alzheimer’s Disease, Friedman Brain Institute, Neuropathology Brain Bank & Research CoRE, Icahn School of Medicine at Mount Sinai, New York, NY USA; ^6^ Department of Pathology, Icahn School of Medicine at Mount Sinai, New York, NY USA; ^7^ James J. Peters Veterans Affairs Medical Center, Bronx, NY USA; ^8^ The Marlene and Paolo Fresco Institute for Parkinson’s and Movement Disorders, NYU Langone Health, New York, NY USA; ^9^ Department of Genetics and Genomic Sciences, Icahn School of Medicine at Mount Sinai, New York, NY USA; ^10^ Icahn Genomics Institute, Icahn School of Medicine at Mount Sinai, New York, NY USA; ^11^ Neural Stem Cell Institute, Rensselaer, NY USA; ^12^ Icahn Institute for Data Science and Genomic Technology, New York, NY USA

## Abstract

**Background:**

Progressive supranuclear palsy (PSP) is the most common primary tauopathy, with a constellation of pathological features including 4R‐tau positive neurofibrillary tangles and tufted astrocytes. Most PSP cases are sporadic and associated with common structural variation in the 17q21.31 *MAPT* locus as well as other loci, including *EIF2AK3* which is critical for the integrated stress response (ISR). Despite these known genetic risk associations, mechanisms underlying disease pathogenesis are unclear. To investigate candidate mechanisms, there is a critical need for model systems that better recapitulate the cellular complexity of the human brain. Induced pluripotent stem cell (iPSC) patient‐derived organoid models are a powerful tool to study molecular and cellular changes in a disease‐relevant genomic context.

**Method:**

Single‐nucleus RNA sequencing (snRNA‐seq) was performed in the subthalamic nucleus region from autopsy PSP and control brains. Transcriptional differences were validated by immunohistochemistry (IHC) using antibodies for ISR activation markers and phosphorylated tau (p‐tau). Fibroblasts grown from sporadic PSP patient skin were reprogrammed into iPSCs, and midbrain organoids were generated in spinner flasks through pharmacological directed differentiation. Total tau, tau isoform, p‐tau, and ISR activation markers were assessed in PSP and control organoids.

**Result:**

Differential gene expression and pathway analysis in PSP brain snRNA‐seq data identified dysregulated EIF2 signaling, a target of the ISR, in vulnerable cell types. Histological validation in autopsy brain tissue showed PSP‐vulnerable brain regions had the highest frequency of ISR activation, while no activation was detected in protected brain regions. ISR activation positively correlated with tau burden and was localized to p‐tau^+^ neurons and astrocytes. PSP organoids contained increased high molecular weight p‐tau and 4R‐tau, a higher ratio of p‐tau:total tau, and different ISR activation levels compared to controls.

**Conclusion:**

SnRNA‐seq and neurohistological data reveals ISR dysregulation in disease‐affected cell types in PSP brain tissue. ISR activation positively associates with tau burden in vulnerable brain regions in PSP, providing a potential mechanistic link with *EIF2AK3* genetic risk. PSP patient‐derived organoids recapitulate key disease‐relevant features, including elevation of toxic tau proteoforms and ISR dysregulation. This sporadic PSP organoid model will provide insight into cell‐type specific drivers of neurodegeneration that underlie sporadic tauopathy.

